# Diagnostic Value of Bronchoalveolar Lavage Fluid Metagenomic Next-Generation Sequencing in *Pneumocystis jirovecii* Pneumonia in Non-HIV Immunosuppressed Patients

**DOI:** 10.3389/fcimb.2022.872813

**Published:** 2022-04-08

**Authors:** He Sun, Feilong Wang, Ming Zhang, Xiaoyong Xu, Miaomiao Li, Wei Gao, Xiaodong Wu, Huize Han, Qin Wang, Gehong Yao, Zheng Lou, Han Xia, Yi Shi, Qiang Li

**Affiliations:** ^1^ Department of Respiratory and Critical Care Medicine, Shanghai East Hospital, School of Medicine, Tongji University, Shanghai, China; ^2^ Department of Respiratory and Critical Care Medicine, The Third Affiliated Hospital of Soochow University, Changzhou, China; ^3^ Department of Pulmonary and Critical Care Medicine (PCCM), The Second Affiliated Hospital of Nanjing University of Chinese Medicine, Nanjing, China; ^4^ Department of Respiratory and Critical Medicine, The Fourth Affiliated Hospital, School of Medicine, Zhejiang University, Yiwu, China; ^5^ Department of Pulmonary and Critical Care Medicine (PCCM), Shenzhen People’s Hospital, The First Affiliated Hospital, Southern University of Science and Technology, Shenzhen, China; ^6^ Department of Respiratory and Critical Care Medicine, Jinling Hospital, Clinical School of Nanjing, Nanjing, China; ^7^ Department of Respiratory and Critical Medicine, Shanghai General Hospital, Shanghai Jiao Tong University School of Medicine, Shanghai, China; ^8^ Department of Scientific Affairs, Hugobiotech, Beijing, China

**Keywords:** metagenomic next-generation sequencing, bronchoalveolar lavage fluid, *Pneumocystis jirovecii* pneumonia, immunosuppressed patients, diagnosis

## Abstract

**Introduction:**

This study aims to assess the value of metagenomic next-generation sequencing (mNGS) of bronchoalveolar lavage fluid (BALF) in the diagnosis of Pneumocystis jirovecii pneumonia (PJP) and its mixed infection in non-human immunodeficiency virus (HIV) immunosuppressed patients.

**Methods:**

A total of 198 non-HIV immunosuppressed patients with severe pneumonia were enrolled, including 77 PJP patients and 121 patients infected by other pathogens. BALF-mNGS and traditional detection methods were used.

**Results:**

The positive detection rate of various pathogens of BALF-mNGS was higher than that of the conventional methods, especially for mixed pathogens. The sensitivity and specificity of BALF-mNGS for the diagnosis of PJP were 97.40% and 85.12%, respectively. Compared with traditional methods, the sensitivity of BALF-mNGS was significantly higher than that of blood fungal G (BG)/lactate dehydrogenase (LDH) and BALF-microscopy (p<0.05), and its specificity was significantly higher than that of BG/LDH (p<0.05). In addition, the average detection time of BALF-mNGS (32.76 ± 10.32 h) was also significantly shorter than conventional methods (p<0.01), especially for mixed infections that were common in non-HIV immunosuppressed patients. In patients only detected as positive by BALF-mNGS, the underlying diseases mainly manifested as hematological malignancies with agranulocytosis and within 8 months after hematopoietic stem cell or solid organ transplantation.

**Conclusions:**

BALF-mNGS technology is faster, more sensitive, and more comprehensive in detecting P. jirovecii and its mixed infection in immunosuppressed patients.

## Introduction

The frequency of community patients with an immunocompromised host has increased dramatically over decades. Despite significant advances in the prevention, diagnosis, and treatment of infection in the immunocompromised host, it remains a major cause of morbidity, increased length of stay, increased total costs, and mortality. Intensive care mortality rates are significantly higher among immunocompromised hosts. The superimposition of the compromised host defenses and critical illness make the detection and management of infections in such patients more difficult but crucial toward salvaging the patient outcome (Shorr et al., 2004; Linden, 2009). Thus, a rapid and accurate pathogen diagnosis is essential to guide anti-infective treatment and improve the prognosis (Cecconi et al., 2018; Fernando et al., 2021). A variety of microbes, including *Pneumocystis jirovecii* and their mixed infections, are important pathogens responsible for severe pneumonia in immunosuppressed patients (Cilloniz et al., 2019). However, the current diagnostic efficiency of conventional methods is merely 30%–40% (Musher et al., 2013; Jain et al., 2015). To improve the efficacy of pathogen diagnosis using quick and accurate test methods is of great importance to the precise anti-infective treatment.

An emerging trend of the increasing incidence of P. jirovecii pneumonia (PJP) has been exhibited recently. This may be due to the increase of various immunodeficient populations [especially in non-human immunodeficiency virus (HIV) immunodeficient patients] (Roux et al., 2014), as well as the improved efficiency of the state-of-the-art technology, metagenomic next-generation sequencing (mNGS), to detect the pathogen (Linden, 2009; Harpaz et al., 2016; Cilloniz et al., 2019; Tsang et al., 2021). It was reported that PJP in non-HIV immunosuppressed patients was characterized by more insidious onset, more rapid progression, and more common concomitant mixed infection with other pathogens (Armstrong-James et al., 2014; Rego de Figueiredo et al., 2019). In addition, the condition was more likely to progress into acute progressive respiratory failure within a short period of time (Kotani et al., 2017). Therefore, early, rapid, and accurate pathogen diagnosis is very important not only to guide the treatment of PJP and mixed infections but also to improve the prognosis.

The state-of-the-art technology, mNGS, can theoretically detect the nucleic acids of all pathogens in the specimens in a rapid, comprehensive, and unbiased manner (Chiu and Miller, 2019). Previous studies have reported that mNGS is able to detect pathogens in a variety of specimens including bronchoalveolar lavage fluid (BALF) (Wilson et al., 2019; Chen et al., 2021; Gu et al., 2021). This technology is characterized by high sensitivity, high accuracy, and short detection time (Chiu and Miller, 2019; Gu et al., 2021). It has significant advantages for diagnosing pathogens responsible for mixed infections in immunosuppressed patients, severe infections, and rare and new pathogen infections (Chiu and Miller, 2019). However, the efficacy of BALF-mNGS in the etiological diagnosis of non-HIV immunosuppressed patients with PJP and mixed infections is unknown.

This study focused on PJP and mixed infections as important types of severe infections in non-HIV-infected immunosuppressed patients. BALF-mNGS was performed to detect PJ and the pathogens of mixed infections. Pathogen diagnostic efficacy and time consumption were analyzed and compared between BALF-mNGS and conventional methods. In addition, the value of BALF-mNGS in the accurate etiological diagnosis of severe pneumonia in non-HIV-infected immunosuppressed patients was also evaluated.

## Materials and Methods

This study complied with the 1964 “Declaration of Helsinki” and its subsequent amendments. This study was approved by the ethics committees of each study institution. Each patient or legal representative provided written informed consent at the time of registration.

### Study Design

The multicenter retrospective study recruited study subjects between May 2017 and May 2021, who were non-HIV immunosuppressed adult patients (age ≥18 years) with community-acquired pneumonia (CAP). The patients were hospitalized in the respiratory intensive care unit (ICU) of 7 general hospitals in Shanghai, Hangzhou, Changzhou, Nanjing, and Shenzhen. CAP and severe community-acquired pneumonia (SCAP) diagnosis met the diagnostic criteria of the Infectious Disease Society of America/American Thoracic Society (Baughman, 2007), excluding patients with Hospital-acquired pneumonia (HAP) or HIV infection. The patients were divided into an observation group (PJP and mixed infection group) and a control group (non-PJP group) according to etiological diagnostic results.

The immunosuppressed state was defined following the previous study (Hill, 2020) as follows: ① hematological malignancies; ② solid tumors chemotherapeutically treated within the past 28 days; ③ hematopoietic stem cell transplantation (HCT) or solid organ transplantation; ④taken antirheumatic drugs, biological immunomodulators, immunosuppressants (e.g., cyclosporin, cyclophosphamide, and methotrexate); ⑤ primary immunodeficiency disease; ⑥ agranulocytosis <1,000/μl; ⑦ taken glucocorticoid daily≥20 mg, lasting ≥14 days (or prednisolone with a cumulative dose >700 mg or equivalent doses of other corticosteroids); ⑧ CD4+ T-lymphocyte counts <200 cells/μl (or percentage <14%).

PJP diagnosis was referred to the criteria of “Chinese guideline for diagnosis and treatment of HIV/AIDS” and “Guideline for the diagnosis of *Pneumocystis jirovecii* pneumonia in patients with hematological malignancies and stem cell transplant recipients” (Alanio et al., 2016; Aids, Hepatitis C Professional Group et al., 2018). The details were as follows: ① non-HIV immunosuppressed hosts; ② with fever or dry cough, shortness of breath; ③ Chest computed tomography (CT) showed multiple ground-glass interstitial exudation, reticulate or consolidated shadows in both lungs; ④ blood/lavage fluid (Linden, 2009; Cecconi et al., 2018) β-D-glucan test (G test) positive (>60 pg/ml) twice; ⑤ elevated peripheral blood lactate dehydrogenase (LDH) (>618 U/L); and ⑥ *P. jirovecii* trophozoites (and/or cysts) were microscopically identified following Wright–Giemsa staining. Clinical diagnosis was made if the aforementioned items ①-⑤ were met, and confirmed diagnosis was made if items ①-⑥ were met.

### Sample Collection and Etiological Diagnosis

BALF was collected according to previous consensus and guideline (Meyer et al., 2012; Diseases CSoR, 2017). After eliminating contraindications, all patients underwent bronchoscopy under intravenous combined anesthesia or 2% lidocaine topical anesthesia. After an equal volume of normal saline was fractionally injected into the affected bronchial segments, BALF was aspirated under negative pressure for related testing.

All patients were subject to pathogen detection using BALF-mNGS and conventional methods at the same time. BALF and peripheral blood specimens were simultaneously submitted for microscopic examination. Lavage fluid was centrifuged to collect the sediment for pathogen identification under the microscope after staining. The fully automated medical PCR analysis system GeneXpert Dx System (Cepheid, Sunnyvale, CA, USA) was used for pathogen nucleic acid detection. A microbial culture (BACT-ALERT; bioMérieux, France) and automated microbial identification systems (VITEK 2 Compact and VITEK MS; bioMérieux, France) were employed.

### Nucleic Acid Extraction, Library Preparation, and Sequencing

DNA was extracted from all samples using a QIAamp^®^ UCP Pathogen DNA Kit (Qiagen), following the manufacturer’s instructions. Human DNA was removed using Benzonase (QIAGEN, Hilden, Germany Sigma, St. Louis, Missouri) and Tween20 (Sigma) (Amar et al., 2021). Libraries were constructed using a Nextera XT DNA Library Prep Kit (Illumina, San Diego, CA, United States) (Miller et al., 2019). The library was quality-assessed by the Qubit dsDNA HS Assay kit, followed by High Sensitivity DNA kit (Agilent) on an Agilent 2100 Bioanalyzer. Library pools were then loaded onto an Illumina Nextseq CN500 sequencer for 75 cycles of single-end sequencing to generate approximately 20 million reads for each library. For negative controls, we also prepared Peripheral blood mononuclear cell (PBMC) samples with 105 cells/ml from healthy donors in parallel with each batch, using the same protocol, and sterile deionized water was extracted alongside the specimens to serve as non-template controls (NTCs) (Miller et al., 2019).

### Bioinformatics Analyses

Trimmomatic was used to remove low-quality reads, adapter contamination, and duplicate reads, as well as those shorter than 50 bp (Bolger et al., 2014). Low-complexity reads were removed by Kcomplexity with default parameters. Human sequence data were identified and excluded by mapping to a human reference genome (hg38) using the Burrows–Wheeler Aligner software (Li and Durbin, 2009). We designed a set of criteria similar to the National Center for Biotechnology Information (NCBI) criteria for selecting the representative assembly for microorganisms (bacteria, viruses, fungi, protozoa, and other multicellular eukaryotic pathogens) from the NCBI Nucleotide and Genome databases. Pathogen lists were selected according to three references: 1) Johns Hopkins ABX Guide (https://www.hopkinsguides.com/hopkins/index/Johns_Hopkins_ABX_Guide/Pathogens), 2) Manual of Clinical Microbiology (Carroll et al., 2019), and 3) clinical case reports or research articles published in current peer-reviewed journals (Fiorini et al., 2017). The final database consisted of about 13,000 genomes. Microbial reads were aligned to the database with SNAP v1.0beta.18. Virus-positive detection results were defined as the coverage of three or more non-overlapping regions on the genome. A positive bacterial or fungal (such as *P. jirovecii*) detection was reported for a given species or genus if the reads per million (RPM) ratio, or RPM-r, was ≥5, where the RPM-r was defined as the RPMsample/RPMNC [i.e., the RPM corresponding to a given species or genus in the clinical sample divided by the RPM in the negative control (NC)]. Additionally, bacterial RPM should be higher than 10 and fungal RPM should be higher than 2 (Miller et al., 2019; Zinter et al., 2019). In addition, to minimize cross-species misalignments among closely related microorganisms, we penalized (reduced) the RPM of microorganisms sharing a genus or family designation, if the species or genus appeared in NTCs. A penalty of 5% was used for species (Gu et al., 2021).

## Results

### Baseline Data

The acquired HIV blood test was negative in all included patients. All patients with malignant tumors have received chemotherapy and/or radiotherapy. The median onset time in transplant patients was 5.82 ± 2.33 months after transplantation. Two HCT patients developed chronic graft-versus-host disease (GVHD). All post-transplant patients received immunosuppressants on a long-term basis, including calmodulin inhibitors, glucocorticoids, and cytokine response inhibitors. Mixed infections by multiple pathogens were common in these non-HIV immunosuppressed patients. The common symptoms of patients with PJP and its mixed infections included fever, dry cough, shortness of breath, and pulmonary rales. The chest CT of PJP patients revealed diffuse ground-glass-like exudates in both lungs. Flalamethoxazole, caspofungin, and methylprednisolone were given for the treatment of PJP, accompanied by other empirical antibiotics for the mixed infections. After treatment, 27 out of 77 PJP patients passed away due to PJP coinfection, complicated with respiratory failure and multiple organ dysfunction; the 30-day case fatality rate reached 36.36%. The patients’ basic clinical data are shown in **Table 1**.

**Table 1:** The basic clinical data of enrolled patients.

	Observation group (n=77)	Control group (n=121)	p-value
**Age (year)**	60.31 ± 19.02	62.02 ± 15.08	>0.05
**Men (%)**	38 (49.35%)	65 (53.71%)	>0.05
**Immunosuppressed diseases**			
Hematological malignancies	23 (29.87%)	8 (6.61%)	<0.01
Solid organ transplantation	14(18.18%)	4 (3.30%)	<0.01
Hematopoietic stem cell transplantation	16 (20.77%)	3 (2.47%)	<0.01
Connective tissue diseases	17 (22.07%)	29 (23.96%)	>0.05
Malignant solid tumors	7 (9.09%)	74 (61.15%)	<0.01
Primary immunodeficient diseases	0 (0)	3 (2.48%)	>0.05
**Time of incidence of PJP after transplantation**			
≤3 months	6 (7.79%)	2 (1.65%)	>0.05
4–12 months	22 (28.57%)	4 (3.30%)	<0.01
>12 months	2 (2.59%)	1 (0.82%)	>0.05
**Application of immunosuppressants**			
Glucocorticoids alone	4 (5.19%)	7 (5.79%)	>0.05
Glucocorticoids+others	36 (46.75%)	29 (23.96%)	<0.01
**Clinical indicators**			
APACH II score	24.90 ± 5.23	25.62 ± 4.98	>0.05
PSI score	152.43 ± 19.32	148.52 ± 17.91	>0.05
Oxygenation index (P/F) mmHg			
P/F>200	9 (11.68%)	46 (38.01%)	<0.01
P/F ≤ 200	68 (88.31%)	75 (61.98%)	<0.01
**Treatments**			
High flow and noninvasive ventilation	41 (53.24%)	86 (71.07%)	<0.05
Intubation and invasive ventilation	36 (46.75%)	35 (28.92%)	<0.05
Vasoconstrictor	33 (42.85%)	41 (33.88%)	>0.05
ECMO	2 (2.59%)	3 (2.47%)	>0.05
**Laboratory indicators**			
Peripheral blood lymphocyte count (×109/L)	0.33 ± 0.27	0.72 ± 0.68	<0.01
Fungal G (BG) test as positive	67 (87.01%)	49 (40.49%)	<0.01
Increased lactate dehydrogenase	71 (92.20%)	57 (47.10%)	<0.01
**30-day mortality**	28 (36.36%)	43 (35.53%)	>0.05

The ratio of patients with hematological malignancies, hematopoietic stem cell transplantation, and solid organ transplantation was significantly higher in the observation group than in the control group (p<0.01), while the ratio of patients with malignant solid tumors in the observation group was significantly lower (p<0.01). In the subgroup of patients receiving transplantation, the incidence of PJP in patients within 4–12 months after transplantation was significantly higher than in the control group (p<0.01). In patients receiving glucocorticoids combined with immunosuppressants, the incidence of PJP was significantly higher than in the control group (p<0.01). This suggests that high-risk factors for PJP may include malignant tumors (regardless of hematological malignancies or solid tumors treated with radiotherapy and chemotherapy), hematopoietic stem cell and solid organ transplantation, and the combined use of multiple immunosuppressants. In addition, the number of patients with PaO2/FiO2 ratio (P/F) ≤200 as well as patients receiving invasive mechanical ventilation and with vasoconstrictor was significantly higher in the observation group than in the control group. This suggests that PJP patients were more susceptible to hypoxic respiratory failure, and with more severe hypoxia, and more PJP patients needed invasive mechanical ventilation and were more prone to shock. Peripheral blood lymphocyte counts were significantly lower in the observation group (p<0.01), suggesting that the declined peripheral blood lymphocyte counts might be a high-risk factor for PJP. The proportion of positive blood fungal G (BG) test and elevated LDH level were significantly higher in the observation group than in the control group (p<0.01), suggesting that both indexes could be used as markers for the preliminary screening of PJP.

### Bronchoalveolar Lavage Fluid–Metagenomic Next-Generation Sequencing Detection

*P. jirovecii* sequences were detected (positive) in 75 of the 77 BALF specimens from the observation group, while other pathogenic sequences were detected in 66 of these specimens by mNGS. The relative abundance of *P. jirovecii* was 37.3%–96.7%. Of these cases, 2 with negative *P. jirovecii* by mNGS were revealed positive by microscopic examination and BALF-PCR. In the control group, *P. jirovecii* sequences were detected in 18 of the 121 BALF specimens, with a relative abundance of 1.2%–3.1%. *P. jirovecii* colonization or contamination was considered.

**Figure 1:**
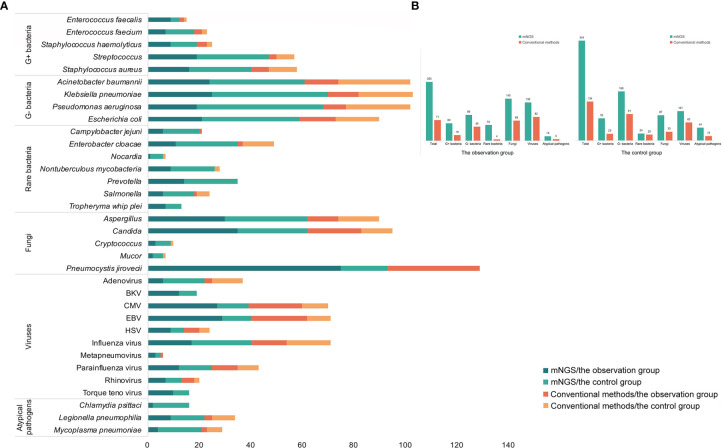
The distribution of detected pathogens of all patients by mNGS and other clinical methods. **(A)** The mNGS results were shown in dark green (the observation group) and green (the control group), and the results of clinical methods were shown in red (the observation group) and orange (the control group). **(B)** The number of each type of pathogens detected by mNGS and clinical methods.

**Figure 2:**
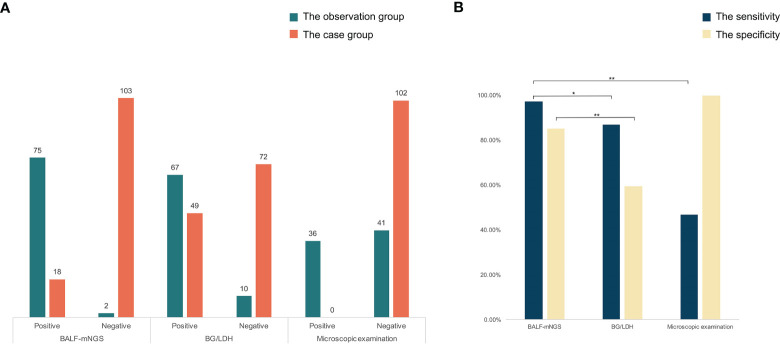
The comparisons of detected results among mNGS, BG/LDH, and microscopic examination. **(A)** The detected result of each patient of the three methods. **(B)** The sensitivity and specificity of the three methods. The sensitivity and specificity of mNGS were significantly higher than the other two methods (p<0.05). p <0.05 was marked as *, and p <0.01 was marked as **.

**Figure 3:**
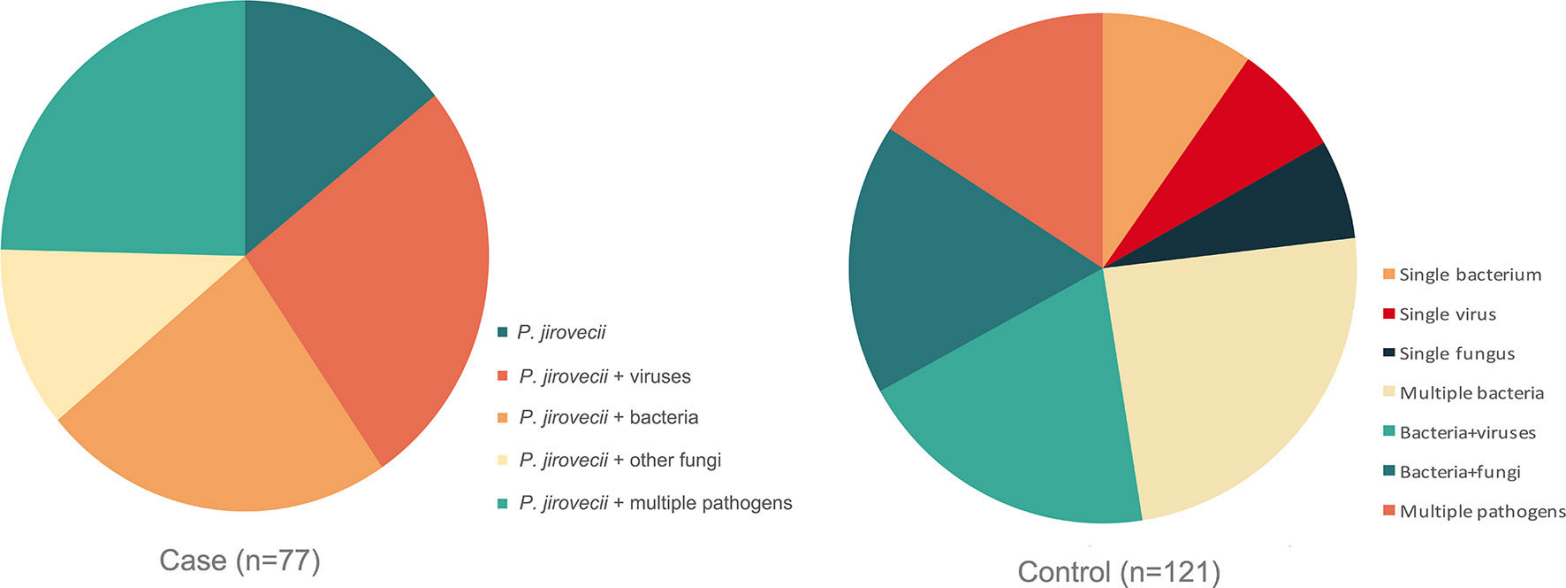
The content of detected pathogens by mNGS in the observation group and control group. Immunosuppressed patients with severe pneumonia were more likely to have mixed infections.

The respiratory pathogen detection results of BALF-mNGS and conventional methods are shown in **Figure 1** and **Table S1**. The positive detection rate of various pathogens was higher using the BALF-mNGS method than using conventional methods in both groups (**Figure 2** and **Table S2**). The contents of pathogens in both groups by BALF-mNGS are shown in **Figure 3** and **Table S3**. Only 14.2% of the observation group patients were with simple *P. jirovecii* infection while most manifested a mixed infection of *P. jirovecii* with other pathogens (85.8%). Viruses (26.3%), especially for CMV and EBV, were the leading pathogens coinfected with *P. jirovecii* in these immunosuppressed patients, followed by bacteria and multiple types of pathogens (**Table S4**). In the control group, mixed infections of multiple pathogens were also common (**Table S5**). Immunosuppressed patients with severe pneumonia were more likely to have mixed infections.

**Figure 4:**
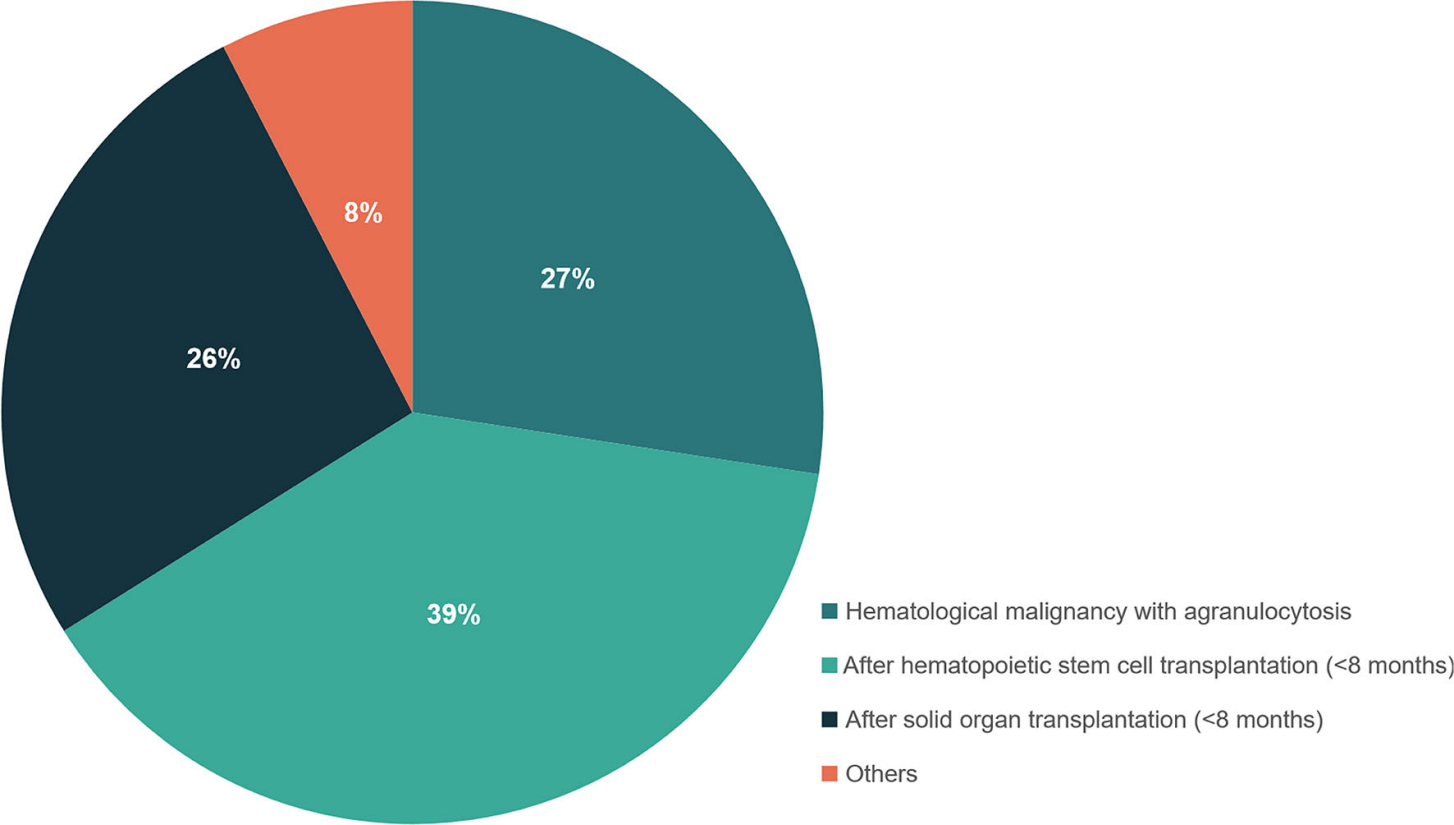
The distribution of different types of underlying diseases in patients with positive mNGS but negative conventional test results. Patients who had hematological malignancies with agranulocytosis, or within 8 months after hematopoietic stem cell transplantation and within 8 months after solid organ transplantation were recommended to perform mNGS detection for suspected infections.

Fifty-nine out of 198 patients had positive BALF-mNGS but negative conventional test results. The distribution of underlying disease types in the 59 patients was shown in **Figure 4** and **Table S6**, with the top three being hematological malignancies with agranulocytosis (27.4%), within 8 months after hematopoietic stem cell transplantation (38.7%) and within 8 months after solid organ transplantation (26.3%). Only 7.6% patients were with other underlying diseases.

### Comparison of BALF-mNGS and Other Diagnostic Methods

Conventional laboratory-based methods, including culture, the G test, BG/LDH, and BALF-microscopy were applied for the diagnosis of *P. jirovecii* and other causative pathogens in each case. In the observation group, BALF-mNGS revealed positive *P. jirovecii* sequences in 75 of the 77 patients; the BG test was positive in 67 cases, accompanied by LDH elevation; and the median serum (Linden, 2009; Cecconi et al., 2018) β-D-glucan was (473.45 ± 159.52) pg/ml, while the median LDH was (954.21 ± 342.01) U/L. However, four *P. jirovecii* BALF-mNGS positive cases were tested negative for the BG test twice. A microscopic examination of BALF identified positive *P. jirovecii* cysts and/or trophozoites in 36 cases (**Supplementary Material**). In the control group, BALF-mNGS revealed positive *P. jirovecii* sequences in 18 of the 121 patients; the BG test was positive in 49 cases, accompanied by LDH elevation; in patients with elevated BG/LDH, the median serum (Linden, 2009; Cecconi et al., 2018) β-D-glucan was (246.43 ± 58.65) pg/ml, while the median of LDH was (978.2 ± 473.12) U/L; and a microscopic examination of BALF showed negative *P. jirovecii* cysts and trophozoites. In the diagnosis of PJP, the sensitivity and specificity of BALF-mNGS, BG/LDH, and microscopic examination were 97.40% and 85.12%, 87.01% and 59.50%, and 46.75% and 100%, respectively (**Figure 3**). BALF-mNGS had significant advantages in the diagnosis of PJP. The average mNGS detection time was (30.54 ± 10.32) h, which was significantly shorter than the conventional laboratory-based diagnostic methods (82.32 ± 30.43) h (p<0.01).

## Discussion

With the increase of immunosuppressed population, severe pneumonia in non-HIV immunosuppressed patients has become a significant part of respiratory intensive care unit (RICU) patients, and the resulting sepsis and multiple organ failure are the important causes of death (Shorr et al., 2004; Ramirez et al., 2020). There are often rare pathogen, conditional pathogen, and multiple pathogen mixed infections due to the impact of underlying diseases and immune function. These infections are characterized by atypical clinical symptoms, rapid disease progression, and high mortality (Linden, 2009; Konduri, 2016). Additionally, these patients often use a variety of anti-infective drugs during the course of disease, resulting in a low pathogen detection rate of conventional methods. Therefore, rapid and accurate pathogen diagnosis has become a clinical challenge. Fortunately, we have this new state-of-the-art technology of mNGS for pathogen identification in severe lung infections. In this study, BALF-mNGS was used in the etiological diagnosis of non-HIV immunosuppressed patients with severe pneumonia admitted to the RICU of 7 hospitals.

Our previous study showed that ~20% patients with severe pneumonia admitted to the RICU were immunosuppressed patients, and *P. jirovecii* was found in 45% of these patients (Wu et al., 2020). In this study, we also found *P. jirovecii* as a major infectious pathogen in immunosuppressed patients, indicating that PJP has become an emerging trend in RICU patients with pneumonia these years. So, dynamically monitoring CD4+ lymphocytes in peripheral blood is necessary for immunosuppressed patients. For immunosuppressed patients with CD4+ lymphocytes <200/μl (<14%), accompanied by fever, shortness of breath, and diffuse ground-glass-like exudates in lungs, *P. jirovecii* and other suspected pathogens should be detected as quickly as possible. The rapid and accurate diagnosis of causative pathogens can help clinicians propose antibiotic treatments, improving the patients’ prognosis.

The ratio of hematological malignancies and transplantation was meaningfully higher in patients with PJP and mixed infection. In 4–12 months after transplantation, the combined use of multiple immunosuppressants was a high-risk factor for PJP. We also found that PJP patients were more susceptible to hypoxic respiratory failure, which was characterized by more severe hypoxia, a higher possibility of receiving invasive mechanical ventilation, and higher susceptibility to shock. This suggested that BALF-mNGS was more urgently needed in these kinds of patients to help guide the subsequent anti-infective treatment.

*P. jirovecii* is an ascomycete, mainly colonizing on the human type I alveolar epithelium (Alanio et al., 2016). It only causes disease in the immunosuppressed population, leading to serous exudative inflammation in the lungs (Lee and Chuang, 2018). The typical lung imaging features of PJP are characterized by diffuse interstitial exudation in both lungs (Alanio et al., 2016; Mu et al., 2016). However, similar imaging findings may be observed in other lung diseases such as lung GVHD, the pulmonary invasion of connective tissue disease, and viral pneumonia (Gotway et al., 2005). Conventional etiological diagnostic methods include microscopic examination, PCR, serum, and/or BALF fungal G test plus serum LDH (Esteves et al., 2015). However, there are some limitations, such as the difficulty of *P. jirovecii* culture *in vitro*, low positive rate of microscopic examination, and high false-positive rate of fungal G tests (Calderon et al., 2010; Huang et al., 2011; Esteves et al., 2014). qPCR has been recommended for the diagnosis of PJP in the European Conference on Infections in Leukaemia (ECIL) guidelines, but it is hard for qPCR to identify all pathogens in immunosuppressed patients with PJP mixed infections, especially for rare and opportunistic pathogens. In addition, there are no commercial qPCR kits for *P. jirovecii* in China, and the cut-off value remains unclear. Nevertheless, qPCR was also performed in some cases of this study, the results were shown in **Table S7** and the **Supplementary Material**. It should be noted that PJP patients with immunosuppression are prone to mixed infections, which made the accurate diagnosis more difficult. A rapid and accurate pathogen diagnosis is needed.

In this study, mNGS showed a higher overall positive detection rate for various pathogens than conventional methods in both groups, especially for PJP mixed with rare and atypical pathogens. This has preliminary illustrated the advantages of BALF-mNGS for rapid pathogen detection in patients with PJP-mixed infections. The 59 severe pneumonia patients with positive BALF-mNGS but negative conventional test results mainly manifested underlying diseases as hematological malignancies with agranulocytosis, within 8 months after hematopoietic stem cell transplantation and within 8 months after solid organ transplantation. This highly suggested that these patients were a population of special concerns and needed early BALF-mNGS diagnosis.

Different from previous studies (Li et al., 2018; Wu et al., 2020), in this study, the vast majority of the observation group were mixed infections of *P. jirovecii* with other pathogens (85.8%), while most patients in the control group also manifested as mixed infections of multiple pathogens (76.9%), suggesting that immunosuppressed patients with severe pneumonia were more prone to mixed infections. To improve the pathogen detection rate, BALF-mNGS should be used as soon as possible despite conventional methods.

Compared with other common pathogens of lung infections such as *Aspergillus*, *Cryptococcus*, and *M. tuberculosis*, the detection rate of *P. jirovecii* using BALF-mNGS was significantly higher. In the same BALF sample, the number of *P. jirovecii* sequences was often higher than other intracellular pathogens. The reason might be that the pathogenic site of *P. jirovecii* is in alveolar epithelial cells, mainly causing diffuse alveolar injuries. Accordingly, conventional cell wall disruption can meet the testing requirements. In addition, given the small amount of sputum in PJP patients, the positive detection rate was higher with lavage fluid than with sputum and blood (Bateman et al., 2020). It suggests that BALF-mNGS can be fully applied in the pathogen diagnosis of PJP. For intracellular pathogens, the positive detection rate is relatively low; therefore, deep wall disruption may be necessary. When intracellular bacterial coinfection is considered, the depth of the wall disruption of the samples should be appropriately increased to improve the overall positive detection rate. Concomitant PCR is also needed to further verify mNGS results. One feasible subplan is to select the tissue samples of the corresponding lesions for mNGS detection with reference to lung HRCT characteristics. Since PJP is prone to the complication of a viral infection, an mNGS RNA detection process is needed. The simultaneous application of mNGS and conventional methods can not only increase the positive rate of pathogen diagnosis but also improve the specificity. In addition, it is necessary to make a comprehensive judgment based on a patient’s clinical characteristics, underlying diseases, imaging characteristics, and background medications to improve the diagnostic accuracy.

Like other highly sensitive test methods, mNGS can also produce false-positive results. The nucleic acid sequences of dead, contaminating, and even locally colonized pathogenic microorganisms are all detectable, sometimes even with a high level of sequence numbers. Sputum specimens and inappropriate lavage specimens are susceptible to the interference of oral and airway-colonizing bacteria, which might increase the background noise and even cause false-positive results. Factors such as sampling sites, standardized sterile operation, specimen submission, and the depth of subsequent wall disruption can also affect the results. Therefore, diagnostic accuracy can further be improved through close cooperation between clinicians and testing microbiologists, through simultaneous mNGS detection and conventional testing of the same specimen.

The pre-evaluation of possible pathogens to carry out targeted detection is the guiding principle for most commonly used conventional detection methods, but it is highly susceptible to the subjective empirical influence of clinical and laboratory personnel, with a narrow pathogen spectrum, leading to a high rate of missed diagnosis. It also requires multiple submissions, a higher specimen demand, and longer detection time. In this study, we found good agreement between BALF-mNGS results and patients’ clinical characteristics in the etiological diagnosis of PJP. The detection spectrum was wider, and the detection time was significantly shorter than conventional methods. It is able to greatly shorten the etiological diagnosis time and provide strong support for rapid and accurate anti-infective treatment.

There were certain limitations for this study. The number of patients enrolled in this study was limited. A prospective study with a larger number of patients would be needed to support the use of mNGS in the early diagnosis of PJP in immunosuppressed patients to further guide the clinical diagnostic standards.

## Data Availability Statement

The datasets presented in this study can be found in online repositories. The names of the repository/repositories and accession number(s) can be found below: http://ngdc.cncb.ac.cn, PRJCA006572.

## Ethics Statement 

All procedures followed were in accordance with the ethical standards of the responsible committee on human experimentation (institutional and national) and with the Helsinki Declaration of 1975, as revised in 2000 (Cilloniz et al., 2019). The patients/participants provided their written informed consent to participate in this study.

## Author Contributions

YS and QL designed the paper and guided the implementation. HS and FW drafted the manuscript. HS, FW, MZ, XX, ML, WG, XW, HH, QW, and GY collected the cases and summarized the data. ZL and HX processed the data and did the statistical analysis. All authors approved the final manuscript as submitted and agree to be accountable for all aspects of the work.

## Funding

This study was supported by The Outstanding Clinical Discipline Project of Shanghai Pudong (PWYgy2018-6) and the Science and Technology Project of Xi'an (No. 21RGSF0013).

## Conflict of Interest

ZL and HX are employed by Hugobiotech Co., Ltd.

The remaining authors declare that the research was conducted in the absence of any commercial or financial relationships that could be construed as a potential conflict of interest.

## Publisher’s Note

All claims expressed in this article are solely those of the authors and do not necessarily represent those of their affiliated organizations, or those of the publisher, the editors and the reviewers. Any product that may be evaluated in this article, or claim that may be made by its manufacturer, is not guaranteed or endorsed by the publisher.
